# Intake and Dietary Food Sources of Fibre in Spain: Differences with Regard to the Prevalence of Excess Body Weight and Abdominal Obesity in Adults of the ANIBES Study

**DOI:** 10.3390/nu9040326

**Published:** 2017-03-25

**Authors:** Liliana G. González-Rodríguez, José Miguel Perea Sánchez, Javier Aranceta-Bartrina, Ángel Gil, Marcela González-Gross, Lluis Serra-Majem, Gregorio Varela-Moreiras, Rosa M. Ortega

**Affiliations:** 1 Department of Nutrition and Dietetics, Faculty of Health Sciences, University Alfonso X El Sabio, Madrid 28691, Spain; liligoro@uax.es (L.G.G.-R.); josepesa@uax.es (J.M.P.S.); 2 VALORNUT Research Group, Department of Nutrition, Faculty of Pharmacy, Complutense University, Madrid 28040, Spain; 3 Department of Preventive Medicine and Public Health, University of Navarra, Pamplona, Navarra 31008, Spain; jaranceta@unav.es; 4 Biomedical Research Networking Center for Physiopathology of Obesity and Nutrition (CIBEROBN), Carlos III Health Institute, Madrid 28029, Spain; agil@ugr.es (A.G.); marcela.gonzalez.gross@upm.es (M.G.-G.); lluis.serra@ulpgc.es (L.S.-M.); 5 Department of Biochemistry and Molecular Biology II and Institute of Nutrition and Food Sciences, University of Granada, Granada 18100, Spain; 6 ImFINE Research Group, Department of Health and Human Performance, Technical University of Madrid, Madrid 28040, Spain; 7 Research Institute of Biomedical and Health Sciences, University of Las Palmas de Gran Canaria, Faculty of Health Sciences, c/Doctor Pasteur s/n Trasera del Hospital, Las Palmas de Gran Canaria, 35016 Las Palmas, Spain; 8 Department of Pharmaceutical and Health Sciences, Faculty of Pharmacy, CEU San Pablo University, Madrid 28668, Spain; gvarela@ceu.es; 9 Spanish Nutrition Foundation (FEN), Madrid 28010, Spain; 10 Department of Nutrition, Faculty of Pharmacy, Madrid Complutense University, Madrid 28040, Spain

**Keywords:** fibre, food sources, obesity, abdominal obesity, misreporting, adults, Spain, ANIBES

## Abstract

The aim was to study the intake and food sources of fibre in a representative sample of Spanish adults and to analyse its association with excess body weight and abdominal obesity. A sample of 1655 adults (18–64 years) from the ANIBES (“Anthropometric data, macronutrients and micronutrients intake, practice of physical activity, socioeconomic data and lifestyles”) cross-sectional study was analysed. Fibre intake and dietary food sources were determined by using a three-day dietary record. Misreporters were identified using the protocol of the European Food Safety Authority. Mean (standard deviation) fibre intake was 12.59 (5.66) g/day in the whole sample and 15.88 (6.29) g/day in the plausible reporters. Mean fibre intake, both in the whole sample and the plausible reporters, was below the adequate intake established by European Food Safety Authority (EFSA) and the Institute of Medicine of the United States (IOM). Main fibre dietary food sources were grains, followed by vegetables, fruits, and pulses. In the whole sample, considering sex, and after adjusting for age and physical activity, mean (standard error) fibre intake (adjusted by energy intake) was higher in subjects who had normal weight (NW) 13.40 (0.184) g/day, without abdominal obesity 13.56 (0.192) g/day or without excess body weight and/or abdominal obesity 13.56 (0.207) g/day compared to those who were overweight (OW) 12.31 (0.195) g/day, *p* < 0.001 or obese (OB) 11.83 (0.266) g/day, *p* < 0.001, with abdominal obesity 12.09 (0.157) g/day, *p* < 0.001 or with excess body weight and/or abdominal obesity 12.22 (0.148) g/day, *p* < 0.001. There were no significant differences in relation with the fibre intake according to the body mass index (BMI), presence or absence of abdominal obesity or excess body weight and/or abdominal obesity in the plausible reporters. Fibre from afternoon snacks was higher in subjects with NW (6.92%) and without abdominal obesity (6.97%) or without excess body weight and/or abdominal obesity (7.20%), than those with OW (5.30%), *p* < 0.05 or OB (4.79%), *p* < 0.05, with abdominal obesity (5.18%), *p* < 0.01, or with excess body weight and/or abdominal obesity (5.21%), *p* < 0.01, in the whole sample. Conversely, these differences were not observed in the plausible reporters. The present study demonstrates an insufficient fibre intake both in the whole sample and in the plausible reporters and confirms its association with excess body weight and abdominal obesity only when the whole sample was considered.

## 1. Introduction

In recent decades, there has been a significant increase in the prevalence of overweight (OW) and obesity (OB) in children and adults, in both developed and developing countries [1]. Excess body weight increases the risk of developing various diseases, such as cardiovascular disease, type 2 diabetes, some types of cancers, musculoskeletal disorders, and neurodegenerative diseases, which have an important health and social impact [1,2,3].

Diet and physical activity are the main factors that influence the development of OW and OB [1,4]. In this respect, several studies have shown that the intake of dietary fibre may have a positive effect on body weight control; however, the available results are inconsistent [5,6,7,8]. There are several possible mechanisms to explain the anti-obesogenic effect of dietary fibre. Among the most documented mechanisms is the one that refers to the benefits of the fibre capacity (mainly soluble fibre) to form viscous gels that delay the gastric emptying, which helps, on the one hand, to increase the satiety sensation and consequently reduces energy intake [9] and, secondly, to control the postprandial glycaemia by delaying the intestinal absorption [10]. Another possible mechanism is associated with short-chain fatty acid produced during fermentation of fibre in the gastrointestinal tract [11] since it has been shown that this can contribute to regulating the secretion of some gastrointestinal hormones, such as glucagon-like peptide-1 (GLP-1), involved in satiety, and ghrelin, involved in appetite control [12,13], and to increase the oxidation of fatty acids and energy expenditure and to regulate glucose metabolism [11].

In addition, there are few recent studies regarding the intake of fibre in a representative Spanish adults sample and none of these studies have analysed the relation between the fibre intake, fibre from different meals of the day and food sources and the problematic of excess body weight and abdominal obesity [14,15]. Additionally, it is the first Spanish study that takes into account the dietary misreporting of the participants.

Therefore, the aim of the present work was to study the intake and dietary food sources of fibre in a representative sample of the Spanish adults from the ANIBES (“Anthropometric data, macronutrients and micronutrients intake, practice of physical activity, socioeconomic data and lifestyles”) study and to analyse the differences in fibre intake between people with different body weight and with or without abdominal obesity. The present work shows the analysed data for the total sample and plausible reporters of the study.

## 2. Materials and Methods

### 2.1. Study Design and Sampling Procedure

The complete design, protocol, and methodology of the ANIBES study have been already described in detail elsewhere [16,17]. In summary, the ANIBES study was carried out to analyse anthropometric data, physical activity, intake of food and beverages, and dietary habits in the Spanish population (9–75 years old, *n* = 2009). The participants were randomly selected from the Northeast, East, Southwest, North-Central, Barcelona, Madrid, Balearic, and Canary Islands areas of Spain, including rural, semi-urban and urban populations [16,17]. The present study is focused on 1655 adults (779 men and 858 women) with ages ranging 18–64.

The following exclusion criteria were applied:

- Those individuals living in an institutional setting (e.g., colleges, nursing homes, hospitals, and others);
- Individuals following a therapeutic diet owing to recent surgery or taking any medical prescriptions;
- Potential participants with a transitory illness (i.e., flu, gastroenteritis) at the time of the fieldwork; and
- Individuals employed in areas related to consumer science, marketing, or the media [16,17].

The fieldwork for the ANIBES study was carried out from mid-September 2013 to mid-November 2013. The final protocol was approved by the Ethical Committee for Clinical Research of the Region of Madrid (Spain) (code FEN 2013/31, May 2013). All participants were informed of the protocol and risks and benefits of their participation in the study and a written informed consent was obtained from all the study’s participants.

### 2.2. Anthropometric Data

Weight, height, and waist circumference (WC) were measured by trained interviewers following standardized procedures [18]. Weight was measured once with a Seca^®^ model 804 weighing scale (Medizinische Messsysteme und Waagen seit 1840, Hamburg, Germany; range: 70–205 cm, precision: 1 mm). Height was assessed in triplicate using a Seca^®^ model 206 stadiometer (Medizinische Messsysteme und Waagen seit 1840, Hamburg, Germany; range: 0.1–150 kg, precision: 100 g). WC was measured in triplicate using a Seca^®^ 201 tape measure (Seca, Hamburg, Germany; range: 0–150 cm, precision: 1 mm).

Excess body weight was assessed using the body mass index (BMI) and abdominal obesity by the waist to height ratio (WHtR). BMI was calculated as weight (kg)/height (m)^2^ and WHtR as WC (cm)/height (cm). Participants were classified into the following categories using the BMI: underweight (UW) (BMI <18.5 kg/m^2^), normal weight (NW) (BMI 18.5–24.9 kg/m^2^), OW (BMI 25–29.9 kg/m^2^), and OB (BMI ≥30 kg/m^2^), based on the World Health Organization classification [19], and using the WHtR respondents were classified into two categories: “non-abdominal obesity” (WHtR <0.5) and those with “abdominal obesity” (WHtR ≥0.5) [20,21,22,23]. BMI and WHtR were combined into one category called “excess body weight and/or abdominal obesity” (BMI ≥25 kg/m^2^ and/or WHtR ≥0.5) for subsequent analysis [19,21,22,23].

### 2.3. Physical Activity

To obtain physical activity data of the studied participants, a detailed interview using the International Physical Activity Questionnaire (IPAQ) for adults was performed [24]. In addition, different physical activity measurements were obtained with an accelerometer ActiGraph (model GT3x and GT3x+; ActiGraph, Pensacola, FL, USA) in a subsample of 167 adults. Individuals were asked to wear the ActiGraph on a belt above the right hip, for three consecutive full days. This previous information was used to validate the physical activity questionnaire administered to the whole sample.

There were calculated the following indicators of physical activity in the participants of the present study:

- Total physical activity expressed as minutes of activity per week;
- Time spent in vigorous physical activity expressed as minutes of activity per week.

Subjects were classified into two levels of physical activity based on two questions of the IPAQ [24]:

“Sedentary activity level” as those subjects who have performed less than 30 min per day of physical activity of daily life and less than 2 h per week of structured physical exercise.

“Active activity level” as those subjects who have performed at least 30 min per day of physical activity of daily life or have performed at least 2 h per week of structured physical exercise.

### 2.4. Food and Beverage Record

Study participants were provided with a tablet device (Samsung Galaxy Tab 2 7.0, Samsung Electronics, Suwon, South Korea) and trained in how to record information by taking photos of all food and beverages consumed during the three days of the study, both at home and outside the home. Photos had to be taken before beginning to eat and drink, and again after finishing, so as to record the actual intake. Additionally, a brief description of meals (including the type of food), recipes, brands, and the use of dietary supplements were recorded using the device.

Energy and fibre intake were calculated from food consumption records using VD-FEN 2.1 software (Dietary Evaluation Programme, Spanish Nutrition Foundation, Madrid, Spain) which was newly developed for the ANIBES study by the Spanish Nutrition Foundation and is based mainly on Spanish food composition tables [25], with several expansions and updates. Data obtained from food manufacturers and nutritional information provided on food labels were also included. A food photographic atlas was also used to assist in assigning gram weights to serving sizes [26].

The present study was focused on the intake of fibre expressed in grams per day (g/day) raw (before the energy intake adjustment) and after the energy intake adjustment by the residual methods [27,28] and the intake of fibre expressed in grams per 1000 kcal per day. The mean intake of fibre of the studied participants was compared with the adequate intake set out by European Food Safety Authority (EFSA): 25 g/day in adults [29], and by the Institute of Medicine of the United States (IOM): 38 g/day for men and 25 g/day for women of 19–50 years and 30 g/day for men and 21 g/day for women of 51–70 years (14 g/1000 kcal/day) [30].

Once fibre intake has been established, the percentage of fibre from each one of the meal times (breakfast, mid-morning snack, lunch, afternoon snack, and dinner) was calculated.

The contribution of each food to total fibre intake has been calculated by adding the amount of fibre provided by each specific food and dividing by the total intake of fibre, all multiplied by 100 [31].

### 2.5. Evaluation of Misreporting

The assessment of dietary intake based on self-reported or self-recorded data by the study subjects is often prone to bias. One of the main sources of error is misreporting, including both under- and over-reporting, which are often influenced by age, sex, and other individual characteristics, including BMI [32]. The magnitude of misreporting of the intake of food may lead to biased interpretation of the results. In the present study the protocol of the EFSA was used to identify misreporters [33,34]. This method is based mainly on the Goldberg [35] and Black [36,37] work. This method evaluates the reported energy intake (EIrep) against the presumed energy requirements. EIrep is expressed as a multiple of the mean basal metabolic rate estimated (BMRest) (from formulas), and it is compared with the presumed energy expenditure of the studied population. Then the ratio EIrep:BMRest is referred to as the physical activity levels (PAL) [33,34]. The PAL is established for adults (≥18 years) in three levels: low, 1.4; moderate, 1.6; and vigorous, 1.8. Additionally, the protocol indicates that analyses should be performed at two levels, group and individual. The group level determines the overall bias to the reported energy intake, and the individual level shows the rate of under and over reporters. The BMRest was calculated using the Schöfield equations [38]. Misreporting cut-offs at group and individual levels for the ANIBES study has been described in a previous paper [39]. Subjects were classified into two categories:

“Plausible reporters” as those subjects with estimated values of the ratio EIrep:BMRest ranging between calculated lower cut-off and upper cut-off values for the studied population allocated to the different category of physical activity [33,34].

“Misreporters” as those subjects with estimated values of EIrep:BMRest below the calculated lower cut-off value (under-reporters) and those subjects with estimated values of EIrep:BMRest above the upper calculated cut -off value (over-reporters) [33,34].

In the present study, the results are showed in the total sample (before the exclusion of the non-plausible reporters) and in the plausible reporters.

### 2.6. Statistical Analysis

Descriptive data were presented as mean and standard deviation (SD), median and interquartile range (IQR) (both without any adjustment), and frequencies (%) stratified by sex, BMI, presence of abdominal obesity, and excess body weight and/or abdominal obesity. The normality of the data and equality of the variances were tested using the Kolmogorov–Smirnov and Levene’s test, respectively.

The statistical differences of the studied quantitative variables by sex were assessed using Student’s *t*-test when data were normally distributed, and using the Mann–Whitney test, otherwise. A two-way ANOVA test was used to assess the differences within the studied variables using sex and the variables of BMI, abdominal obesity or excess body weight and/or abdominal obesity. Two-way ANOVA main effects for BMI, abdominal obesity, and excess body weight and/or abdominal obesity were obtained. The Bonferroni post hoc test for comparison for more than two groups was used. A two-way ANCOVA test was used to assess the differences within the studied variables using the previous ANOVA model taking into account the age and the physical activity (PA) of the participants as covariates to control their influence on the dependent variable. Two-way ANCOVA main effects and interactions were analysed and they are only reported in the text as estimated marginal means and the standard error (SE) when a significant inverse trend, significant changes, or an interaction with sex were observed. The level of significance was set at *p* < 0.05. All calculations were performed using IBM SPSS version 22.0 (IBM Corp., Armonk, NY, USA).

## 3. Results

### 3.1. Study Population

A sample of 1655 adults (48.2% men and 51.8% women) with ages ranging from 18 to 64 years was studied. In total, 26.16% (*n* = 433) (36.4% men and 63.5% women) of the studied adults were classified as plausible reporters. Table 1 shows the anthropometric and physical activity data of the whole sample and of the plausible reporters. The combined prevalence of OW and OB in the whole sample was 55.70% and 32.80% in the plausible reporters. The prevalence of abdominal obesity was 58.40% and 39.3% and the presence of excess body weight and/or abdominal obesity was 63.93% and 42.7% in the whole sample and in the plausible reporters, respectively. A 44.40% of the whole sample and 44.3% of the plausible reporters were sedentary.

### 3.2. Fibre Intake and Dietary Food Sources in the Whole Sample and in the Plausible Reporters and by Sex

Mean dietary fibre intake (raw) was 12.59 (SD 5.66) g/day, and adjusted by energy was 12.59 (SD 4.91) g/day, while the intake of fibre expressed as grams per 1000 kcal per day was 7.05 (SD 2.81) in the whole sample (Table 2). Fibre intake (raw) was significantly lower in women than in men, but when it was adjusted by energy intake or when was expressed as grams per 1000 kcal per day, it was significantly lower in men than in women in the whole sample (Table 2). Meanwhile, in the group of plausible reporters, the mean dietary fibre (raw) was 15.88 (SD 6.29) g/day, and it was also significantly lower in women than in men, however, there were no statistically significant differences in relation with the fibre intake adjusted by the energy intake or expressed as g/1000 kcal/day (Table 2). Lunch and dinner contributed the highest proportions of fibre to the total daily intake in the whole sample and in the plausible reporters (75.85% and 70.59%, respectively). It is noteworthy that the fibre from breakfast was quite low in the whole sample (13.04%) as well as in the plausible reporters (14.27%). The proportion of fibre from breakfast and afternoon snacks was higher in women and from dinner in men in the whole sample. No significant differences were observed regarding the fibre from the different meals in the plausible reporters (Table 2).

The main food sources of fibre in the whole studied population and in the plausible reporters were grains (39.13% and 39.14%), vegetables (24.17% and 20.99%), fruits (16.60% and 18.51%), pulses (9.28% and 9.14%), ready-to-eat-meals (4.50% and 4.99%), sauces and condiments (2.18% and 1.97%), appetizers (1.53% and 2.30%), sugars and sweets (0.67% and 1.15%), non-alcoholic beverages (0.49% and 0.53%), dairy products (0.39% and 0.28%), and supplements and meal replacers (0.14% and 0.09%, respectively).

### 3.3. Fibre Intake and Dietary Food Sources in the Whole Sample and in the Plausible Reporters by Body Mass Index Classification

Considering the whole sample, the fibre intake (raw) was higher in subjects who had NW compared to those who had OW or OB, taking into account sex and after adjusting for the age and PA (Table 3). When the interaction of sex by BMI was analysed, it was noted that the intake of fibre (raw) was slightly different according to sex. Men who presented NW 14.83 (SE 0.327) g/day had a higher intake compared to those who had OW 12.45 (SE 0.304) g/day (*p* < 0.001) or OB 11.67 (SE 0.413) g/day (*p* < 0.001). While women who were UW 14.52 (SE 1.077) g/day had a higher intake than those with OW 11.48 (SE 0.335) g/day (*p* < 0.05) or OB 11.33 (SE 0.455) g/day (*p* < 0.05).

Fibre intake adjusted by energy intake (considering sex) was similar for the different groups of BMI in the whole sample (Table 3). However, after adjusting for the age and the PA, it was observed that subjects with NW 13.29 (SE 0.183) g/day had a higher intake than subjects with OW 12.26 (SE 0.194) g/day (*p* < 0.001) or OB 11.79 (SE 0.265) g/day (*p* < 0.001). Analysing the interaction of sex at different levels of BMI, these differences were observed only in men. Thus, men who presented NW 13.46 (SE 0.279) g/day had a higher intake in comparison with those who had OW 11.81 (SE 0.260) g/day (*p* < 0.001) or OB 11.33 (SE 0.353) g/day (*p* < 0.001).

The intake of fibre per 1000 kcal per day (considering sex) was similar for the different groups of BMI in the whole sample (Table 3). However, after adjusting for the age and the PA, it was observed that subjects with NW 7.35 (SE 0.104) g/1000 kcal/day had a higher intake than subjects with OW 6.92 (SE 0.110) g/1000 kcal/day (*p* < 0.05) or OB 6.68 (SE 0.151) g/1000 kcal/day (*p* < 0.01).

No significant differences were observed regarding the fibre intake (raw, adjusted by the energy intake and expressed as grams per 1000 kcal/day) according with the BMI in the plausible reporters, considering sex and after adjusting for the age and the PA (Table 3).

The proportion of fibre from the lunch was higher in individuals with OW than those with NW taking into account sex, but this difference disappeared when an adjustment for age and PA was performed. Fibre from the afternoon snack was higher in individuals with NW than those with OW or OB (taking into account sex and even after adjusting for age and PA) (Table 3). While fibre from dinner was lower (after adjusting for the age and PA) in individuals who had OW 27.50% (SE 0.581) compared to those with OB 30.27% (SE 0.795) (*p* < 0.05). However, the proportion of fibre from the different meals was similar according the BMI in the plausible reporters (Table 3).

Regarding the fibre food sources according to the BMI in the whole sample, we observed that fibre from breakfast cereals and cereal bars was higher in individuals with NW than those with OW and fibre from vegetables was higher in individuals with OB than those with NW, but these differences disappeared when an adjustment for age and PA was performed (Table S1). Fibre from fruits (taking into account sex and after adjusting for the age and PA) was significantly higher in individuals with NW 18.36% (SE 0.574%) compared to subjects with OW 15.16% (SE 0.605%) (*p* < 0.01). In analysing the interaction of sex, it was observed that only in men, the fibre from fruits was higher in those with NW 18.50% (SE 0.881%) than those with OW 14.01% (SE 0.819%) (*p* < 0.001) or OB 12.92% (SE 1.094%) (*p* < 0.001).

After excluding misreporters, we observed that fibre from grains and flours was higher in subjects with NW than those subjects with OW, but this difference disappeared when an adjustment for age and PA was performed (Table S2). Fibre from pasta (taking into account sex and after adjusting for age and PA) was significantly higher individuals with UW 10.83 (SE 2.231%) compared to subjects with NW 4.72% (0.383%) (*p* < 0.05) or OW 4.59% (0.580%) (*p* < 0.05). In analysing the interaction of sex, it was observed that only in men, the fibre from pasta was higher in those with UW 17.98% (SE 4.207%) than in those with NW 4.71% (SE 0.616%) (*p* < 0.05) or OW 4.62% (SE 0.835%) (*p* < 0.05).

### 3.4. Fibre Intake and Dietary Food Sources in the Whole Sample and in the Plausible Reporters by the Presence of Abdominal Obesity Determined by the Waist to Height Ratio

Having into account the whole sample, fibre intake (raw) was higher in subjects without abdominal obesity (taking into account sex and even after adjusting for the age and PA) than those with abdominal obesity (Table 4). In analysing the interaction of sex was found that fibre intake in both men and women without abdominal obesity was higher 15.14 (SE 0.337) g/day and 12.76 (SE 0.276) g/day than those subjects with abdominal obesity 12.04 (SE 0.244) g/day (*p* < 0.001) and 11.47 (SE 0.264) g/day (*p* < 0.01), respectively.

Fibre intake (adjusted by energy intake) considering the sex of the participants was similar independently of the presence or absence of abdominal obesity in the whole sample (Table 4). However, after adjusting for the age and PA, subjects without abdominal obesity 13.43 (SE 0.191) g/day had significantly higher intake than subjects with abdominal obesity 12.05 (SE 0.157) g/day (*p* < 0.001). In analysing the interaction of sex was found that fibre intake in both men and women without abdominal obesity was higher 13.63 (SE 0.288) g/day and 13.24 (SE 0.236) g/day that those with abdominal obesity 11.57 (SE 0.208) g/day (*p* < 0.001) and 12.52 (SE 0.226) g/day (*p* < 0.05), respectively.

The intake of fibre per 1000 kcal per day (considering sex) was similar regardless the presence or absence of abdominal obesity in the whole sample (Table 4). However, after adjusting for the age and the PA, it was observed that the subjects without abdominal obesity 7.44 (SE 0.109) g/1000 kcal/day had a higher intake than subjects with abdominal obesity 6.79 (SE 0.089) g/1000 kcal/day (*p* < 0.001). An interaction of sex was found and it was observed that fibre intake only in men without abdominal obesity was higher 7.36 (SE 0.164) g/1000 kcal/day than in those men with abdominal obesity 6.40 (SE 0.119) g/1000 kcal/day (*p* < 0.001).

Nevertheless, no significant differences were observed regarding the fibre intake (raw, adjusted by the energy intake and expressed as grams per 1000 kcal/day) according with the presence or absence of abdominal obesity, considering sex and after adjusting for the age and the PA in the plausible reporters (Table 4).

Fibre from the mid-morning snack was higher in subjects without abdominal obesity than in subjects with abdominal obesity in the whole sample, but this difference disappeared when an adjustment for age and PA was performed (Table 4).

Considering sex and after adjusting for age and PA, the proportion of fibre from lunch was higher in subjects with abdominal obesity than those without abdominal obesity. In contrast, fibre from the afternoon snack was higher in individuals without abdominal obesity unlike those with abdominal obesity (Table 4). The proportion of fibre from the different meals was similar according the presence or absence of abdominal obesity in the plausible reporters (Table 4).

Concerning the fibre food sources according to the presence or absence of abdominal obesity in the whole sample, we observed that fibre from grains, breakfast cereals, and cereal bars, ready-to-eat meals, and sauces and condiments was higher in individuals without abdominal obesity than those with abdominal obesity and fibre from vegetables was higher in individuals with abdominal obesity than those subjects without abdominal obesity, but all of these differences disappeared when an adjustment for age and PA was performed (Table S3). We only found that fibre from bread (taking into account sex and after adjusting for the age and PA) was higher in subjects with abdominal obesity than those who did not have this problem (Table S3). Similarly, after adjusting for the age and the PA, it was observed that the fibre from pasta was higher in individuals with abdominal obesity 6.73% (SE 0.272%) unlike those participants without abdominal obesity 5.68% (SE 0.332%) (*p* < 0.05). In contrast, the fibre from fruits (taking into account sex and after adjusting for the age and PA) was higher in subjects without abdominal obesity 18.46% (SE 0.600%) than in subjects with abdominal obesity 15.46% (SE 0.491%) (*p* < 0.001). However, when the interaction with sex was analysed (Table S3), the previous result only was observed in men without abdominal obesity 19.15% (SE 0.902%) compared to those with abdominal obesity 13.78% (SE 0.654%) (*p* < 0.001). In relation to the group of sugars and sweets and specifically chocolates, individuals without abdominal obesity had a higher intake than subjects with abdominal obesity (Table S3).

In the plausible reporters, we observed that fibre from pasta was higher in subjects without abdominal obesity than those with abdominal obesity, but this difference disappeared when an adjustment for age and PA was performed (Table S4). Fibre from ready-to-eat-meals (taking into account sex and after adjusting for age and PA) was significantly higher in individuals with abdominal obesity 6.39% (SE 0.585%) compared to subjects without abdominal obesity 4.02% (0.484%) (*p* < 0.01).

### 3.5. Fibre Intake and Dietary Food Sources in the Whole Sample and in the Plausible Reporters by the Presence of Excess Body Weight and/or Abdominal Obesity Using the Body Mass Index and Waist to Height Ratio

Fibre intake (raw) was higher in individuals without excess body weight and/or abdominal obesity in the whole sample (taking into account sex and after adjusting for the age and PA) (Table 5). An interaction of sex was found and it was observed that fibre intake in both men and women without excess body weight and/or abdominal obesity was higher 15.22 (SE 0.371) g/day and 12.81 (SE 0.291) g/day than in those who had excess body weight and/or abdominal obesity 12.28 (SE 0.232) g/day (*p* < 0.001) and 11.55 (SE 0.252) g/day (*p* < 0.01), respectively.

Fibre intake (adjusted by energy intake) considering sex was similar according to the presence or absence of excess body weight and/or abdominal obesity in the whole sample (Table 5). However, after adjusting for the age and PA, it was found that subjects without excess body weight and/or abdominal obesity 13.43 (SE 0.206) g/day had significantly higher intake than subjects with excess body weight and/or abdominal obesity 12.18 (SE 0.148) g/day (*p* < 0.001). An interaction of sex was found and it was observed that fibre intake only in men without excess body weight and/or abdominal obesity was higher 13.68 (SE 0.317) g/day than in those men with excess body weight and/or abdominal obesity 11.74 (SE 0.198) g/day (*p* < 0.001).

The intake of fibre per 1000 kcal per day (considering sex) was similar regardless the presence or absence of excess body weight and/or abdominal obesity in the whole sample (Table 5). However, after adjusting for the age and the PA, it was observed that the subjects without excess body weight and/or abdominal obesity 7.43 (SE 0.117) g/1000 kcal/day had a higher intake than subjects with excess body weight and/or abdominal obesity 6.86 (SE 0.084) g/1000 kcal/day (*p* < 0.001). An interaction of sex was found and it was observed that fibre intake only in men without excess body weight and/or abdominal obesity was higher 7.36 (SE 0.180) g/1000 kcal/day than in those men with excess body weight and/or abdominal obesity 6.48 (SE 0.113) g/1000 kcal/day (*p* < 0.001).

No significant differences were observed regarding the fibre intake (raw, adjusted by the energy intake and expressed as grams per 1000 kcal/day) according with the presence or absence of excess body weight and/or abdominal obesity in the plausible reporters, considering sex and after adjusting for the age and the PA (Table 5).

Considering the sex and after adjusting for the age and the PA, the proportion of fibre from lunch was higher in subjects with excess body weight and/or abdominal obesity than those who did not have excess body weight and/or abdominal obesity. In contrast, the fibre from the afternoon snack was higher in individuals who had no excess body weight and/or abdominal obesity, unlike those with excess body weight and/or abdominal obesity (Table 5). The proportion of fibre from the different meals was similar according the presence or absence of excessive body weight and/or abdominal obesity in the plausible reporters (Table 5).

Relating to the fibre food sources, according to the presence or absence of excess body weight and/or abdominal obesity in the whole sample, we found that fibre from grains, breakfast cereals, and cereal bars, chocolates, ready-to-eat meals, sauces, and condiments, was higher in individuals without excess body weight and/or abdominal obesity than those with excess body weight and/or abdominal obesity, and fibre from vegetables was higher in individuals with excess body weight and/or abdominal obesity than those subjects without excess body weight and/or abdominal obesity, but all these differences disappeared when an adjustment for age and PA was performed (Table S5). Fibre from bread (taking into account sex and after adjusting for the age and PA) was higher in subjects who had excess body weight and/or abdominal obesity than in those who did not have this problematic (Table S5). Similarly, after adjusting for the age and the PA, it was observed that the fibre from the pasta was higher in individuals with excess body weight and/or abdominal obesity 6.65% (SE 0.256%) as opposed to those without it 5.63% (SE 0.358%) (*p* < 0.05). In contrast, fibre from fruits (taking into account sex and after adjusting for the age and PA) was higher in subjects without excess body weight and/or abdominal obesity 19.06% (SE 0.644%) than in subjects with excess body weight and/or abdominal obesity 15.45% (SE 0.462%) (*p* < 0.001). However, when analysing the interaction with sex (Table S5), this result was only observed in men without excess body weight and/or abdominal obesity 20.12% (SE 0.990%) compared to those with excess body weight and/or abdominal obesity 13.87% (SE 0.619%) (*p* < 0.001).

In the plausible reporters, we observed that fibre from the groups of grains and flours, pasta and juices and nectars was higher in subjects without excess body weight and/or abdominal obesity than those with excess body weight and/or abdominal obesity, but these differences disappeared when an adjustment for age and PA was performed (Table S6). Taking into account sex and after adjusting for age and PA, fibre from fruits was significantly higher in individuals without excess body weight and/or abdominal obesity 20.2% (SE 1.042%) compared to subjects with excess body weight and/or abdominal obesity 16.9% (1.144%) (*p* < 0.05). Conversely, the fibre from ready-to-eat-meals was higher in those subjects with excess body weight and/or abdominal obesity 6.41 (SE 0.558%) than those without excess body weight and/or abdominal obesity 3.81% (SE 0.508%) (*p* < 0.01).

## 4. Discussion

The present study provides updated information on fibre intake and dietary sources and their association with the condition of excess body weight and abdominal obesity in a representative sample of the Spanish adult population. It is highlighted the ANIBES is the first national diet and nutrition survey in Spain that has taken into account the plausible reporters in the analysis of the data, based on well-harmonised procedures [33,34].

A great proportion of participants of the whole sample and of the plausible reporters had OW or OB, which is in concordance with other studies performed in the Spanish population [40,41]. Nonetheless, it is highlighted the prevalence of OW or OB was lower in the plausible reporters in comparison with the whole sample. When comparing our results with the FANPE study (carried out in 2009 on a representative sample of the Spanish population), we found that the combined prevalence of OW and OB of the study (47.80%) was lower than that observed in our study in the whole sample (55.70%) and higher than that observed in the plausible reporters (32.80%) [41], while the combined prevalence observed in the ENPE study, conducted in 2014–2015, also in Spain, was higher (60.9%) than that observed in the present study both in the whole sample and in the plausible reporters [40].

Some studies have indicated that the WHtR is a better predictor of metabolic syndrome or cardiovascular disease and mortality than the WC or BMI [21,23]. Possibly, the most important advantage of using the WHtR resides in the fact that this ratio takes into account the height of the subject, which avoids the overestimation or underestimation of individuals who have a high or low height [21,23,42]. In our study, the mean WHtR in the whole sample was 0.52 (SD 0.08) and 0.49 (SD 0.07) in the plausible reporters. These values are in line to those indicated by the FANPE and ENPE studies [40,43]. Using this parameter, 58.4% of the whole sample and 39.30% of the plausible reporters had abdominal obesity, lower than those indicated in the DARIOS Study (conducted in 2013 in Spanish population), in which 89% of men and 77% of women had abdominal obesity [44]. Moreover, when the presence of excess body weight and/or the presence of abdominal obesity were examined, it was found that 63.93% of the whole sample and 42.70% of the plausible reporters had one or both of these problems.

Furthermore, we found that a high proportion of the studied population was sedentary, which has been already discussed in detail in a previous paper [45].

The mean dietary fibre intakes (raw, adjusted by energy intake and expressed in grams per 1000 kcal per day) both in the whole sample and in the plausible reporters were very similar and were lower in comparison with the observed in other studies performed in population with similar ages and characteristics [14,15,46,47]. When comparing the results of our study with those observed by the ENIDE study (carried out in a representative sample of the Spanish population aged 18 to 64 years in 2011), the fibre intake of the participants of our study was lower than the results of such study (men: 20.94 (SD 11.38) g/day and women: 18.85 (SD 10.06) g/day) [14]. Likewise, the mean fibre intakes shown in other study also performed in Spain 20.2 (SD 7.8) g/day and in a study carried out in Irish population 25.7 (SD 8.1) g/day were higher than that observed in our study [15,46]. However, our results were more similar to those observed in the National Health and Nutrition Examination Survey (NHANES 2009–2010) performed in United States adults aged 19 years and older where the fibre intake was 17.0 g/day [47].

The mean intake of fibre (raw) both in men and women was below the adequate intake established by the EFSA and IOM [29,30] in the whole sample and in the plausible reporters. This situation was close to the results shown in various studies performing in similar population [14,46,48,49].

We observed only in the whole sample that the fibre intake after adjusting for energy intake and the fibre per 1000 kcal per day were significantly higher in women than in men. This may be explained because women are usually more concerned about following healthy eating habits and tend to include more healthy foods in their diet compared to men [50]. The analysis of the source of daily fibre intake depending on the different meals throughout the day, revealed that nearly half came from lunch (47.46% and 42.98%) and almost a third from dinner (28.39% and 27.61%) in the whole sample and plausible reporters, respectively. It is noteworthy that the fibre from breakfast was too low in the whole sample (13.04%) and in the plausible reporters (14.27%). The number of frequency of meals per day, including snacks, has been positively related to the intake of various nutrients including the fibre intake [51]. In our study, the mid-morning and afternoon snacks provided the 11.08% and the 15.13% of the daily fibre in the whole sample and in the plausible reporters, respectively. In contrast to our results, the 2001–2010 NHANES study observed that most of the daily fibre came from dinner (37% for adults 19–50 years) [52]. Furthermore, the pattern of fibre intake from the different meals of the day differs according to sex. The proportion of fibre from breakfast and afternoon snacks was higher in women and from dinner in men only in the whole sample. These differences in the pattern of fibre intake are probably due to the differences in the food choices made by the subjects at each meal of the day [52]. A better contribution of fibre from breakfast or afternoon snack, with respect to other meals, could help to reduce appetite and food intake at subsequent meals [6,9]. This could be related to the better situation observed in women compared to men in connection with the presence of excess body weight and abdominal obesity described in a previous paper in more detail for the ANIBES Spanish adult population [53].

Regarding dietary sources of fibre, the main sources in the studied sample were grains, followed by vegetables, fruits, and pulses and were very similar in both studied samples. Similar food groups were identified as the major contributors in the Belgian population [49]. Unlike our study, the main sources of fibre in the ENIDE study were fruits (30%), followed by legumes and nuts (26%), cereals (22%), and vegetables (14%) [14]. Differences were also observed when compared to the results of the 2001–2010 NHANES study, where it was observed that the main sources were the vegetables, followed by cereals and fruits [52].

Some studies performed on populations from the United States [5], the Netherlands [4], and Spain [54] have observed an inverse association between fibre intake and BMI. In accordance with this, in our study, taking into account sex, and after adjusting for the age and physical activity of the participants, fibre intake (raw, adjusted by energy intake and expressed as grams per 1000 kcal per day) was different according to the BMI only in the whole sample. Participants with NW had a significantly higher intake of fibre (raw and expressed as grams per 1000 kcal per day) than those subjects with OW or OB. However, the differences on fibre intake adjusted by the energy intake only were observed in the male sex, where men with NW had a greater intake than those who had OW or OB, which is consistent with what is stated in the study conducted in the Dutch population, where they have found an inverse association between fibre intake and BMI only in men [4]. Moreover, the pattern of fibre intake in the different meals during the day varied depending on the body weight situation only in the whole sample. Specifically, we found that the percentage of fibre that comes from the afternoon snack was higher in individuals with NW than those with OW or OB, while the fibre from dinner was higher in individuals who had OB than those who had OW. The difference found regarding the contribution of fibre from the afternoon snack according to BMI, as previously mentioned, could be related, on the one hand, to the fact that a higher fibre content may favour a reduced appetite which, in turn, can help to take in less food at subsequent meals, in this case during dinner, thus balancing the daily energy intake [6,9]. On the other hand, this also could be explained due to an afternoon snack that contains a higher amount of fibre, which could also include healthier foods with a lower content of energy or fat.

An inverse relation between dietary fibre from cereals and fruits and body weight gain has been described in a study performed in male adults (40–75 years old) from the United States [55]. Although the fibre from grains was the most important fibre food source in the present study, there were no significant differences according to BMI in the whole sample. Conversely, we found that the intake of fibre coming from fruits was higher in men with NW compared to those who had OW or OB in the whole sample. These differences may be explained due to fruits that are rich in soluble fibre, which may help control appetite, or because people who consume fruits and vegetables regularly also tend to have a healthier lifestyle [9,49].

We only observed in the men of the group of plausible reporters that fibre from pasta was higher in those with UW than in those with NW or OW. This may be due to subjects who have an excessive body weight try to control their weight by the reduction of carbohydrates of the diet reducing the intake of this type of food.

When the data were analysed according to the presence or absence of abdominal obesity using the WHtR, we found that the intake of fibre (raw, adjusted by energy intake and express per 1000 kcal per day) only in the whole sample was higher in those subjects without abdominal obesity. In this manner, it becomes clear that the fibre intake may help to avoid the appearance of abdominal obesity, which has also been described in a study performed in Chinese adults that observed subjects with a lower WHtR had a higher fibre intake [56].

On the other hand, the pattern of fibre intake in the different meals during the day varied depending on the presence or absence of abdominal obesity only in the whole sample. The proportion of fibre from lunch was higher in the participants with abdominal obesity compared to their normal counterparts, which could be due to the style of eating in Spain, since in general, lunch is characterized by the presence of an abundant amount of foods like cereals, pulses and vegetables that provide a great amount of fibre, respect to other meals of the day. This type of lunch is largely respected by the general population regardless of individual food habits. However, in relation to the snack and dinner, in Spain is observed that there is a much more marked difference in the composition depending on the food habits of each person. On the other hand, the proportion of fibre from afternoon snacks was higher in individuals without abdominal obesity than in those who had this problem. This finding suggests that a higher intake of fibre in the afternoon could have a beneficial effect in relation to the abdominal accumulation of body fat and not only with respect to the body weight situation described previously. Nevertheless, further studies are needed to clarify this aspect.

Some studies have indicated that the consumption of whole grain foods seems to have benefits regarding weight control and abdominal adiposity, emphasizing that the consumption of whole grain products seems to have not promote weight gain, while refined-grain products are directly associated with the excess of weight and abdominal fat [7,57]. In our study, the contribution to the intake of fibre from bread and pasta was higher only in subjects with abdominal obesity of the whole sample. However, the information about the type of bread or pasta consumed by the population was not available in our study. Moreover, various studies have found an inverse association between dietary fibre from fruits and the WC, insulin resistance, and metabolic syndrome [58,59,60]. In line with this, in our study, the fibre from fruits was higher only in men of the whole sample without abdominal obesity than in those with this problem. This difference, as previously noted, could be explained because of the beneficial effect of soluble fibre on the reduction of the appetite or due to a healthier lifestyle [9,49]. A similar trend was also observed in relation to the group of sugars and sweets and, in particular, with the subgroup of chocolates. However, because the food groups disaggregated by food items have not been analysed, it was not possible to give an explanation. However, it is assumed that this difference may be due to participants without abdominal obesity selecting and consuming some type of chocolate or similar healthier product with a higher content of fibre, unlike the group who has abdominal obesity.

We only observed in the group of plausible reporters that fibre from ready-to-eat-meals was higher in individuals with abdominal obesity compared to subjects without abdominal obesity, which is according with following an unhealthy diet rich in ready-to-eat-meals, which is common in individuals with obesity.

Likewise, when analysing the excess body weight and abdominal obesity individually, it was confirmed that there was a greater fibre intake in subjects without excess body weight and abdominal obesity compared with those who had one or two of these problems only in the whole sample.

It is noted that the fibre from the afternoon snack seems to play a major role in the body weight situation and abdominal obesity. Even when considering the presence or absence of both problems in the same individual, we also found that those subjects in the whole sample with excess body weight or abdominal obesity had a lower proportion of fibre from the afternoon snack and higher from lunch than their counterparts. This suggests that, probably, the meal of the day in which the fibre is consumed is of relevance to obtaining the benefits of the fibre in relation to excess body weight or abdominal obesity.

In relation to the dietary fibre sources according to the excess body weight and/or abdominal obesity, we found a consistent tendency when the sources were analysed according to the excess body weight and abdominal obesity separately. A higher proportion of fibre from bread and pasta (in men and woman), and less from fruit (only in men), is associated with excess body weight and/or abdominal obesity in comparison with subjects with NW and without abdominal obesity.

As in the whole sample, in the plausible reporters we also observed that fibre from fruits was significantly higher in individuals without excess body weight and/or abdominal obesity compared to subjects with excess body weight and/or abdominal obesity. However, only in the plausible reporters, the fibre from ready-to-eat-meals was higher in those subjects with excess body weight and/or abdominal obesity than those without excess body weight and/or abdominal obesity, which is consistent with the results when the fibre dietary sources were analysed according to the presence or absence of abdominal obesity individually.

A limitation of our study was the inability to analyse the types of fibre (soluble and insoluble) and the food groups disaggregated by food items, since such information was not available. Nonetheless, this did not represent an impediment to achieve the aim of our work that was to study the intake and dietary food sources of fibre and analyse the differences in the fibre intake between people with different body weight situations, and with or without abdominal obesity. In contrast, the main strengths of our study include the methodological design used in the ANIBES study, such as the fact that all anthropometric data were measured and they were not self-reported by the participants, which improves the validity of the study, and the possibility of extrapolating our results to the Spanish population because it was conducted in a representative sample. It is important to highlight that this is the first Spanish study at national level that analyses the data for the whole population and the plausible reporters.

The findings regarding the association between diet and the health outcomes analysed in the present study should be interpreted with caution given the discrepancy observed between both samples. Further studies considering different methods to address misreporting are needed to confirm the association between the fibre intake and the excess body weight and/or abdominal obesity. At the same time, the information derived from our study can be useful in designing nutrition intervention strategies to increase the intake of fibre in our country that it was low both in the whole sample and in the plausible reporters, which in turn could prevent and control some health problems such as excess body weight and abdominal obesity.

## 5. Conclusions

The present study demonstrates an insufficient fibre intake among the Spanish adult population, both in the whole sample and in the plausible reporters. The main dietary sources were grains, followed by vegetables, fruits, and pulses for both samples. This study observed an association between the fibre intake and excess body weight and abdominal obesity in the whole sample but not in the plausible reporters. Further studies are needed to confirm the association between the fibre intake and the excess body weight and/or abdominal obesity. Nonetheless, it is advisable to increase the intake of foods rich in fibre in order to prevent diseases related with a low intake.

## Figures and Tables

**Table 1:** Anthropometric and physical activity data in the whole sample and in plausible reporters of the ANIBES study of the Spanish adult population (18–64 years).

Personal, Anthropometric and Physical Activity Data	Statistical Data	Whole Sample	Plausible Reporters
*n*		1655	433
Age (years)	Mean (SD)	39.97 (12.19)	38.53 (11.67)
	P50 (IQR)	39.00 (20.00)	37.00 (17.00)
Weight (kg)	Mean (SD)	74.18 (16.47)	65.71 (13.06)
	P50 (IQR)	72.10 (21.80)	63.80 (17.2)
Height (cm)	Mean (SD)	167.67 (9.35)	165.98 (9.56)
	P50 (IQR)	167.00 (14.00)	165.00 (14.00)
Body mass index (kg/m^2^)	Mean (SD)	26.31 (5.14)	23.78 (3.88)
	P50 (IQR)	25.60 (6.20)	23.10 (5.00)
Underweight	%	1.80	4.60
Normal	%	42.50	62.60
Overweight	%	35.80	25.20
Obesity	%	19.90	7.60
Waist circumference (cm)	Mean (SD)	88.06 (14.50)	81.42 (11.70)
	P50 (IQR)	86.83 (19.74)	79.73 (16.17)
Waist to height ratio	Mean (SD)	0.52 (0.08)	0.49 (0.07)
	P50 (IQR)	0.51 (0.11)	0.48 (0.09)
Non-abdominal obesity	%	41.60	60.70
Abdominal obesity	%	58.40	39.30
Non-excess body weight and/or abdominal obesity	%	36.07	57.30
Excess body weight and abdominal obesity	%	63.93	42.70
Physical activity (minutes/week)	Mean (SD)	856.60 (637.72)	889.22 (640.23)
	P50 (IQR)	735.00 (920.00)	800.00 (960.00)
Vigorous activity (minutes/week)	Mean (SD)	149.20 (264.15)	168.68 (275.13)
	P50 (IQR)	0 (180.00)	0 (260)
Sedentary activity level	%	44.40	44.30
Active activity level	%	55.60	55.70

SD: standard deviation; P50: 50th percentile; IQR: interquartile range; Abdominal obesity: waist to height ratio ≥ 0.5; Excess body weight and/or abdominal obesity: body mass index ≥25 kg/m^2^ and/or waist to height ratio ≥ 0.5.

**Table 2:** Fibre intake in the whole sample and in plausible reporters of the ANIBES study of the Spanish adult population (18–64 years) by sex.

**Whole Sample**	**Statistical Data**	**Total (*n* = 1655)**	**Men (*n* = 798)**	**Women (*n* = 857)**	***p***
Energy (kcal/day)	Mean (SD)	1815.58 (511.96)	1966.22 (543.22)	1675.31 (436.85)	***
	P50 (IQR)	1758.00 (683)	1919.00 (770)	1648.00 (598)	
Total fibre (g/day) (raw)	Mean (SD)	12.59 (5.66)	13.09 (6.09)	12.13 (5.20)	**
	P50 (IQR)	11.63 (6.98)	12.15 (7.27)	11.27 (6.68)	
Total fibre (g/day) (adjusted by energy intake)	Mean (SD)	12.59 (4.91)	12.26 (5.14)	12.91 (4.67)	***
	P50 (IQR)	11.75 (6.01)	11.32 (5.79)	12.25 (6.00)	
Fibre per 1000 kcal/day	Mean (SD)	7.05 (2.81)	6.71 (2.66)	7.37 (2.91)	***
	P50 (IQR)	6.55 (3.53)	6.25 (3.24)	6.99 (3.78)	
Fibre from breakfast (%)	Mean (SD)	13.04 (11.59)	12.15 (11.68)	13.88 (11.44)	***
	P50 (IQR)	10.84 (15.02)	9.73 (15.68)	11.78 (14.48)	
Fibre from mid-morning snack (%)	Mean (SD)	5.16 (8.19)	4.99 (8.21)	5.31 (8.18)	NS
	P50 (IQR)	7.51 (11.69)	0.00 (8.01)	0.87 (8.20)	
Fibre from lunch (%)	Mean (SD)	47.46 (16.60)	48.00 (16.83)	46.96 (16.37)	NS
	P50 (IQR)	47.10 (22.74)	47.61 (23.11)	46.17 (22.50)	
Fibre from afternoon snack (%)	Mean (SD)	5.92 (8.67)	5.48 (8.94)	6.34 (8.39)	***
	P50 (IQR)	1.52 (9.66)	0 (8.55)	3.13 (10.62)	
Fibre from dinner (%)	Mean (SD)	28.39 (14.05)	29.35 (14.43)	27.49 (13.64)	*
	P50 (IQR)	27.03 (18.67)	27.82 (19.40)	26.53 (18.49)	
**Plausible Reporters**	**Statistical Data**	**Total (*n* = 433)**	**Men (*n* = 158)**	**Women (*n* = 275)**	***p***
Energy (kcal/day)	Mean (SD)	2352 (425)	2712 (358)	2145 (306)	***
	P50 (IQR)	2297 (625)	2640 (490)	2095 (403)	
Total fibre (g/day) (raw)	Mean (SD)	15.88 (6.29)	17.97 (7.19)	14.68 (5.36)	***
	P50 (IQR)	14.89 (7.38)	16.63 (8.26)	14.10 (7.40)	
Total fibre (g/day) (adjusted by energy intake)	Mean (SD)	15.88 (5.81)	15.93 (6.87)	15.85 (5.12)	NS
	P50 (IQR)	14.78 (7.69)	14.26 (8.38)	14.94 (7.46)	
Fibre per 1000 kcal/day	Mean (SD)	6.79 (2.44)	6.64 (2.52)	6.87 (2.38)	NS
	P50 (IQR)	6.31 (3.36)	6.07 (3.03)	6.47 (3.46)	
Fibre from breakfast (%)	Mean (SD)	14.27 (11.39)	13.68 (10.89)	14.61 (11.66)	NS
	P50 (IQR)	12.04 (13.54)	11.75 (13.88)	12.45 (12.90)	
Fibre from mid-morning snack (%)	Mean (SD)	6.32 (8.35)	5.99 (7.91)	6.50 (8.60)	NS
	P50 (IQR)	3.38 (9.32)	2.53 (9.44)	3.78 (9.28)	
Fibre from lunch (%)	Mean (SD)	42.98 (14.84)	44.05 (15.06)	42.37 (14.70)	NS
	P50 (IQR)	42.22 (20.90)	43.32 (21.27)	41.05 (22.17)	
Fibre from afternoon snack (%)	Mean (SD)	8.81 (9.66)	8.16 (9.76)	9.19 (9.60)	NS
	P50 (IQR)	6.15 (13.72)	5.05 (12.22)	6.46 (13.25)	
Fibre from dinner (%)	Mean (SD)	27.61 (12.52)	28.12 (12.11)	27.32 (12.76)	NS
	P50 (IQR)	26.01 (17.25)	27.04 (16.31)	25.79 (18.58)	

SD: standard deviation; P50: 50th percentile; IQR: interquartile range; *p*: denotes statistical mean differences by sex. * *p* < 0.05. ** *p* < 0.01. *** *p* < 0.001. NS: non-significant.

**Table 3:** Fibre intake in the whole sample and in the plausible reporters of the ANIBES study of the Spanish adult population (18–64 years) by body mass index.

**Whole Sample**	**Statistical Data**	**Underweight (*n* = 30)**	**Normal Weight (*n* = 704)**	**Overweight (*n* = 592)**	**Obesity (*n* = 329)**	**Two-Way ANOVA**	**Two-Way ANCOVA**
Energy intake (kcal/day)	Mean (SD)	1930.90 (592.45)	1872.51 (526.97)	1773.45 (502.33) _b_	1759.05 (475.65) _b_	S ** BMI ***	S ** BMI ***
	P50 (IQR)	1857.00 (2114.00)	1827.00 (4018.00)	1712.50 (2843.00)	1676.00 (3109.00)		
Total fibre intake (g/day) (raw)	Mean (SD)	12.86 (6.35)	13.05 (5.98)	12.27 (5.40) _b_	12.19 (5.33) _b_	BMI ** I *	BMI *** I *
	P50 (IQR)	11.20 (22.98)	11.82 (40.66)	11.46 (34.16)	11.46 (35.71)		
Total fibre (g/day) (adjusted by energy) intake)	Mean (SD)	12.22 (4.56)	12.73 (5.13)	12.50 (4.71)	12.50 (4.83)	NS	BMI *** I *
	P50 (IQR)	11.19 (7.68)	11.86 (6.19)	11.90 (6.07)	11.57 (5.81)		
Fibre per 1000 kcal/day	Mean (SD)	6.58 (2.39)	7.06 (2.78)	7.05 (2.79)	7.08 (2.97)	S *	S * BMI **
	P50 (IQR)	6.15 (9.10)	6.56 (18.82)	6.64 (18.35)	6.42 (23.84)		
Fibre from breakfast (%)	Mean (SD)	10.51 (11.89)	13.25 (11.56)	13.16 (11.79)	12.65 (11.30)	NS	NS
	P50 (IQR)	7.34 (41.49)	11.01 (69.25)	10.94 (61.66)	10.37 (64.58)		
Fibre from mid-morning snack (%)	Mean (SD)	6.29 (9.91)	5.32 (8.00)	5.19 (8.48)	4.64 (7.93)	NS	NS
	P50 (IQR)	0.29 (35.40)	1.08 (58.11)	0 (58.29)	0 (63.99)		
Fibre from lunch (%)	Mean (SD)	47.19 (15.69)	45.83 (16.24	48.99 (16.87) _b_	48.25 (16.71)	BMI **	NS
	P50 (IQR)	46.20 (57.35)	44.81 (93.52)	48.96 (88.61)	48.18 (97.88)		
Fibre from afternoon snack (%)	Mean (SD)	7.43 (5.93)	6.92 (9.11)	5.30 (8.49) _b_	4.79 (7.99) _b_	BMI **	BMI *
	P50 (IQR)	6.80 (19.78)	3.25 (52.26)	0.0 (66.44)	0.0 (60.28)		
Fibre from dinner (%)	Mean (SD)	28.58 (12.39)	28.66 (13.96)	27.35 (14.06)	29.65 (14.32)	NS	BMI *
	P50 (IQR)	29.00 (66.69)	27.95 (79.03)	25.80 (91.30)	27.64 (76.37)		
**Plausible Reporters**	**Statistical Data**	**Underweight (*n* = 20)**	**Normal Weight (*n* = 271 )**	**Overweight (*n* = 109)**	**Obesity (*n* = 33)**	**Two-Way ANOVA**	**Two-Way ANCOVA**
Energy intake (kcal/day)	Mean (SD)	2153 (466)	2307 (425)	2443 (394) _a,b_	2539 (395) _a,b_	BMI **	S *** BMI ***
	P50 (IQR)	2070 (799)	2244 (619)	2405 (616)	2413 (459)		
Total fibre intake (g/day) (raw)	Mean (SD)	14.12 (6.35)	15.58 (6.35)	16.69 (5.86)	16.75 (6.97)	NS	NS
	P50 (IQR)	11.96 (10.08)	14.62 (7.13)	16.45 (8.34)	15.82 (6.45)		
Total fibre (g/day) (adjusted by energy) intake)	Mean (SD)	15.25 (4.93)	15.83 (5.81)	16.17 (5.84)	15.69 (6.41)	NS	NS
	P50 (IQR)	14.24 (8.93)	14.89 (7.09)	15.48 (8.71)	14.31 (7.62)		
Fibre per 1000 kcal/day	Mean (SD)	6.44 (2.19)	6.78 (2.44)	6.93 (2.46)	6.59 (2.48)	NS	NS
	P50 (IQR)	6.15 (4.26)	6.38 (3.12)	6.53 (3.96)	6.08 (3.26)		
Fibre from breakfast (%)	Mean (SD)	11.81 (13.33)	14.47 (11.63)	14.12 (10.64)	14.69 (10.81)	NS	NS
	P50 (IQR)	8.03 (19.27)	12.23 (14.34)	13.08 (12.56)	13.45 (14.12)		
Fibre from mid-morning snack (%)	Mean (SD)	9.07 (11.13)	6.47 (8.57)	6.10 (7.86)	4.14 (5.30)	NS	NS
	P50 (IQR)	6.05 (16.17)	3.78 (9.31)	2.64 (9.42)	1.71 (7.15)		
Fibre from lunch (%)	Mean (SD)	42.40 (15.06)	42.61 (15.11)	43.72 (14.52)	43.97 (14.00)	NS	NS
	P50 (IQR)	42.29 (29.63)	40.16 (20.22)	45.93 (21.66)	43.65 (22.68)		
Fibre from afternoon snack (%)	Mean (SD)	8.12 (6.06)	8.60 (9.28)	9.58 (11.37)	8.43 (8.50)	NS	NS
	P50 (IQR)	6.66 (10.27)	6.15 (13.48)	5.27 (14.07)	7.79 (12.94)		
Fibre from dinner (%)	Mean (SD)	28.61 (12.83)	27.85 (12.69)	26.48 (11.89)	28.77 (13.26)	NS	NS
	P50 (IQR)	26.64 (16.05)	26.68 (17.34)	25.48 (16.86)	26.58 (18.43)		

SD: standard deviation; P50: 50th percentile; IQR: interquartile range; Two-way ANOVA was performed taking into account sex (S) and body mass index (BMI); Two-way ANCOVA was performed taking into account S and BMI and age and physical activity as covariates; Two-way ANOVA significant differences between BMI classification: a: regarding underweight, b: regarding normal weight, c: regarding overweight; I: Interaction; * *p* < 0.05, ** *p* < 0.01, *** *p* < 0.001; NS: non-significant.

**Table 4:** Fibre intake in the whole sample and in plausible reporters of the ANIBES Study Spanish adult population (18–64 years) by the presence or absence of abdominal obesity using the waist to height ratio.

**Whole Sample**	**Statistical Data**	**Non-Abdominal Obesity (*n* = 689)**	**Abdominal Obesity (*n* = 966)**	**Two-Way ANOVA**	**Two-Way ANCOVA**
Energy intake (kcal/day)	Mean (SD)	1886.03 (543.28)	1765.33 (482.43)	S *** WHtR ***	S *** WHtR ***
	P50 (IQR)	1832.00 (701.00)	1705.00 (653.00)		
Total fibre intake (g/day) (raw)	Mean (SD)	12.96 (5.97)	12.33 (5.43)	S *** WHtR ** I **	S *** WHtR *** I **
	P50 (IQR)	11.94 (7.34)	11.46(6.52)		
Total fibre (g/day) (adjusted by energy intake)	Mean (SD)	12.58 (5.06)	12.61 (4.81)	I *	WHtR *** I **
	P50 (IQR)	11.71 (6.06)	11.81 (5.83)		
Fibre per 1000 kcal/day	Mean (SD)	6.97 (2.80)	7.11 (2.82)	S ***	S *** WHtR *** I *
	P50 (IQR)	6.46 (3.54)	6.59 (3.49)		
Fibre from breakfast (%)	Mean (SD)	13.15 (11.81)	12.97 (11.43)	S **	S *
	P50 (IQR)	10.75 (15.44)	10.87 (15.04)		
Fibre from mid-morning snack (%)	Mean (SD)	5.70 (8.59)	4.77 (7.88)	WHtR *	NS
	P50 (IQR)	1.44 (8.23)	0 (7.80)		
Fibre from lunch (%)	Mean (SD)	45.35 (16.26)	48.97 (16.67)	WHtR ***	WHtR **
	P50 (IQR)	44.29 (23.29)	48.81(22.57)		
Fibre from afternoon snack (%)	Mean (SD)	6.97 (8.96)	5.18 (8.38)	WHtR ***	WHtR **
	P50 (IQR)	3.31 (11.75)	0 (8.03)		
Fibre from dinner (%)	Mean (SD)	28.81 (14.26)	28.09 (13.91)	S **	S *
	P50 (IQR)	27.96 (18.71)	26.64 (18.59)		
**Plausible Reporters**	**Statistical Data**	**Non-Abdominal Obesity (*n* = 263)**	**Abdominal Obesity (*n* = 170)**	**Two-Way ANOVA**	**Two-Way ANCOVA**
Energy intake (kcal/day)	Mean (SD)	2319.06 (446.79)	2401.78 (384.418)	S ***	S ***
	P50 (IQR)	2244.00 (641.00)	2366.50 (558)		
Total fibre intake (g/day) (raw)	Mean (SD)	15.36 (6.28)	16.67 (6.23)	S ***	S ***
	P50 (IQR)	14.47 (7.36)	16.32 (7.67)		
Total fibre (g/day) (adjusted by energy intake)	Mean (SD)	15.54 (5.57)	16.39 (6.13)	NS	NS
	P50 (IQR)	14.65 (7.01)	15.27 (8.23)		
Fibre per 1000 kcal/day	Mean (SD)	6.63 (2.31)	7.03 (2.59)	NS	NS
	P50 (IQR)	6.22 (3.21)	6.52 (3.59)		
Fibre from breakfast (%)	Mean (SD)	14.36 (11.94)	14.12 (10.49)	NS	NS
	P50 (IQR)	11.89 (14.42)	13.16 (12.53)		
Fibre from mid-morning snack (%)	Mean (SD)	6.88 (9.00)	5.43 (7.14)	NS	NS
	P50 (IQR)	4.11 (10.44)	2.60 (8.64)		
Fibre from lunch (%)	Mean (SD)	41.97 (14.99)	44.54 (14.50)	NS	NS
	P50 (IQR)	39.91 (19.86)	45.79 (23.04)		
Fibre from afternoon snack (%)	Mean (SD)	8.57 (9.10)	9.18 (10.48)	NS	NS
	P50 (IQR)	6.28 (13.48)	6.03 (13.92)		
Fibre from dinner (%)	Mean (SD)	28.19 (12.64)	26.70 (12.31)	NS	NS
	P50 (IQR)	26.87 (17.32)	25.50 (16.84)		

SD: standard deviation; P50: 50th percentile; IQR: interquartile range; Two-way ANOVA was performed taking into account sex (S) and the waist to height ratio (WHtR); Two-way ANCOVA was performed taking into account S and the WHtR and the age and physical activity as covariates. I: interaction. * *p* < 0.05. ** *p* < 0.01. *** *p* < 0.001. NS: non-significant.

**Table 5:** Fibre intake in the whole sample and in plausible reporters of the ANIBES study of the Spanish adult population (18–64 years) by the presence or absence of excess body weight and/or abdominal obesity using the body mass index and the waist to height ratio.

**Whole Sample**	**Statistical Data**	**Non-Excess Body Weight and/or Abdominal Obesity (*n* = 597)**	**Excess Body Weight and/or Abdominal Obesity (*n* = 1058)**	**Two-Way ANOVA**	**Two-Way ANCOVA**
Energy intake (kcal/day)	Mean (SD)	1892.54 (539.47)	1772.15 (490.72)	S *** BMI_WHtR ***	S *** BMI_WHtR ***
	P50 (IQR)	1848.00 (686.00)	1707.00 (669.00)		
Total fibre intake (g/day) (raw)	Mean (SD)	13.00 (5.96)	12.36 (5.49)	S *** BMI_WHtR** I**	S *** BMI_WHtR *** I**
	P50 (IQR)	11.94 (7.25)	11.47 (6.54)		
Total fibre (g/day) (adjusted by energy intake)	Mean (SD)	12.58 (5.09)	12.60 (4.81)	I *	BMI_WHtR *** I **
	P50 (IQR)	11.71 (6.14)	11.79 (5.86)		
Fibre per 1000 kcal/day	Mean (SD)	6.97 (2.78)	7.1 (2.84)	S ***	S *** BMI_WHtR ** I *
	P50 (IQR)	6.47 (3.56)	6.59 (3.5)		
Fibre from breakfast (%)	Mean (SD)	13.21 (11.85)	12.96 (11.45)	S * I *	S * I *
	P50 (IQR)	10.76 (15.27)	10.85 (15.07)		
Fibre from mid-morning snack (%)	Mean (SD)	5.66 (8.31)	4.88 (8.13)	NS	NS
	P50 (IQR)	1.46 (8.31)	0 (7.76)		
Fibre from lunch (%)	Mean (SD)	45.17 (16.17)	48.76 (16.71)	BMI_WHtR ***	BMI_WHtR **
	P50 (IQR)	44.13 (22.98)	48.64 (22.37)		
Fibre from afternoon snack (%)	Mean (SD)	7.20 (9.09)	5.21 (8.35)	BMI_WHtR ***	BMI_WHtR **
	P50 (IQR)	3.61 (12.03)	0 (8.03)		
Fibre from dinner (%)	Mean (SD)	28.76 (13.84)	28.19 (14.18)	S **	S *
	P50 (IQR)	27.91 (18.71)	26.79 (18.62)		
**Plausible Reporters**	**Statistical Data**	**Non-Excess Body Weight and/or Abdominal Obesity (*n* = 248)**	**Excess Body Weight and/or Abdominal Obesity (*n* = 185)**	**Two-Way ANOVA**	**Two-Way ANCOVA**
Energy intake (kcal/day)	Mean (SD)	2296 (434)	2426 (402)	S ***	S *** BMI_WHtR **
	P50 (IQR)	2223 (623)	2385 (601)		
Total fibre intake (g/day) (raw)	Mean (SD)	15.16 (6.09)	16.85 (6.43)	S ***	S ***
	P50 (IQR)	14.42 (7.27)	16.36 (7.87)		
Total fibre (g/day) (adjusted by energy intake)	Mean (SD)	15.47 (5.47)	16.43 (6.20)	NS	NS
	P50 (IQR)	14.62 (6.84)	15.33 (8.33)		
Fibre per 1000 kcal/day	Mean (SD)	6.62 (2.31)	7.02 (2.58)	NS	NS
	P50 (IQR)	6.22 (3.10)	6.53 (3.71)		
Fibre from breakfast (%)	Mean (SD)	14.34 (12.12)	14.19 (10.35)	NS	NS
	P50 (IQR)	11.47 (14.64)	13.31 (12.24)		
Fibre from mid-morning snack (%)	Mean (SD)	6.98 (9.08)	5.43 (7.17)	NS	NS
	P50 (IQR)	4.22 (10.74)	2.63 (8.70)		
Fibre from lunch (%)	Mean (SD)	41.93 (14.99)	44.40 (14.56)	NS	NS
	P50 (IQR)	39.75 (20.10)	45.69 (22.85)		
Fibre from afternoon snack (%)	Mean (SD)	8.71 (9.22)	8.96 (10.25)	NS	NS
	P50 (IQR)	6.37 (13.86)	5.98 (13.44)		
Fibre from dinner (%)	Mean (SD)	28.05 (12.81)	27.02 (12.13)	NS	NS
	P50 (IQR)	26.71 (17.24)	25.64 (17.04)		

SD: standard deviation; P50: 50th percentile; IQR: interquartile range; Two-way ANOVA was performed taking into account sex (S) and the body mass index and/or waist to height ratio (BMI-WHtR); Two-way ANCOVA was performed taking into account S and the BMI-WHtR and the age and physical activity as covariates. I: interaction. * *p* < 0.05. ** *p* < 0.01. *** *p* < 0.001. NS: non-significant.

## Supplementary Materials

The following are available online at www.mdpi.com/1999-4907/9/04/325/s1, Table S1: Dietary food sources of total fibre (%) in the whole sample of the ANIBES study of the Spanish adult population (18–64 years) by body mass index., Table S2: Dietary food sources of total fibre (%) in the plausible reporters of the ANIBES study of the Spanish adult population (18–64 years) by body mass index, Table S3: Dietary food sources of total fibre (%) in the whole sample of the ANIBES Study Spanish adult population (18–64 years) by the presence or absence of abdominal obesity using the waist to height ratio, Table S4: Dietary food sources of total fibre (%) in the plausible reporters of the ANIBES Study Spanish adult population (18–64 years) by the presence or absence of abdominal obesity using the waist to height ratio, Table S5: Dietary food sources of total fibre (%) in the whole sample of the ANIBES study of the Spanish adult population (18–64 years) by the presence or absence of excess body weight and/or abdominal obesity using the body mass index and the waist to height ratio, Table S6: Dietary food sources of total fibre (%) in the plausible reporters of the ANIBES study of the Spanish adult population (18–64 years) by the presence or absence of excess body weight and/or abdominal obesity using the body mass index and the waist to height ratio.

## Abbreviations

ANIBES	Anthropometric data, macronutrients and micronutrients intake, practice of physical activity, socioeconomic data and lifestyles
BMI	Body Mass Index
EFSA	European Food Safety Authority
GLP-1	Glucagon-like peptide-1
IOM	Institute of Medicine of the United States
IPAQ	International Physical Activity Questionnaire
NHANES	National Health and Nutrition Examination Survey
NW	Normal weight
OB	Obesity
OW	Overweight
PA	Physical activity
UW	Underweight
WC	Waist circumference
WHtR	Waist to height ratio
