# Supplementary material for: Intake and Dietary Food Sources of Fibre in Spain: Differences with Regard to the Prevalence of Excess Body Weight and Abdominal Obesity in Adults of the ANIBES Study
Source: Nutrients. 2017 Mar 25;9(4):326. doi: 10.3390/nu9040326 (PMC5409665; doi:10.3390/nu9040326)
Supplement: Supplementary file 1 [file nutrients-09-00326-s001.docx]

Supplementary Materials: Intake and Dietary Food Sources of Fibre in Spain. Differences with Regard to the Prevalence of Excess Body Weight and Abdominal Obesity in Adults of the ANIBES Study

Liliana G. González-Rodríguez, José Miguel Perea Sánchez, Javier Aranceta-Bartrina, Ángel Gil, Marcela González-Gross, Lluis Serra-Majem, Gregorio Varela-Moreiras, Rosa M Ortega

**Table S1.** Dietary food sources of total fibre (%) in the whole sample of the ANIBES study of the Spanish adult population (18–64 years) by body mass index.

|  | **Underweight**  **(n = 30)** | **Normal**  **(n = 704)** | **Overweight**  **(n = 592)** | **Obesity**  **(n = 329)** | **Two-way ANOVA** | **Two-way**  **ANCOVA** |
| --- | --- | --- | --- | --- | --- | --- |
| Grains | 42.19 | 39.85 | 38.89 | 37.77 | NS | NS |
| Grains and flours | 3.97 | 3.80 | 3.41 | 2.75 | NS | NS |
| Bread | 22.88 | 20.30 | 21.46 | 21.60 | NS | NS |
| Breakfast cereals and cereal bars | 1.52 | 1.92 | 1.25 _b_ | 1.19 | BMI* | NS |
| Pasta | 4.83 | 6.45 | 6.06 | 6.44 | NS | NS |
| Bakery and pastry | 9.80 | 8.17 | 7.72 | 6.50 | S* | S* |
| Vegetables | 20.53 | 23.14 | 24.54 | 26.02 _b_ | BMI** | NS |
| Fruits | 12.88 | 16.74 | 16.00 | 17.70 | I* | BMI* I* |
| Oils and fats | - | - | - | - |  |  |
| Milk and dairy products | 0.30 | 0.39 | 0.37 | 0.44 | NS | NS |
| Yogurt and fermented milk | 0.16 | 0.31 | 0.32 | 0.35 | NS | NS |
| Other dairy products | 0.14 | 0.08 | 0.05 | 0.09 | NS | NS |
| Fish and Shellfish | - | - | - | - |  |  |
| Meat and meat products | - | - | - | - |  |  |
| Eggs | - | - | - | - |  |  |
| Pulses | 11.38 | 9.21 | 9.63 | 8.58 | NS | NS |
| Sugars and sweets | 2.19 | 0.75 | 0.61 | 0.49 | S* | S* |
| Chocolates | 1.62 | 0.53 | 0.39 | 0.25 | S* | S* |
| Jams and other | 0.57 | 0.18 | 0.18 | 0.14 | S* | S* |
| Other sweets | 0.00 | 0.04 | 0.04 | 0.10 | NS | NS |
| Appetizers | 1.43 | 1.54 | 1.61 | 1.36 | NS | NS |
| Ready-to-eat-meals | 5.79 | 4.72 | 4.33 | 4.20 | NS | NS |
| Sauces and condiments | 2.04 | 2.24 | 2.22 | 2.01 | NS | NS |
| Non-alcoholic beverage | 0.45 | 0.52 | 0.54 | 0.35 | NS | NS |
| Juices and nectars | 0.45 | 0.49 | 0.48 | 0.30 | NS | NS |
| Other drinks | 0.00 | 0.02 | 0.05 | 0.05 | NS | NS |
| Supplements and meal replacers | 0.00 | 0.09 | 0.14 | 0.26 | NS | NS |

Two-way ANOVA was performed taking into account sex (S) and body mass index (BMI); Two-way ANCOVA was performed taking into account S and BMI and age and physical activity as covariates; Two way ANOVA significant differences between BMI classification: a: regarding underweight, b: regarding normal weight, c: regarding overweight; I: Interaction; * *p* < 0.05, ** *p* < 0.01. NS: non-significant.

**Table S2.** Dietary food sources of total fibre (%) in the plausible reporters of the ANIBES study of the Spanish adult population (18–64 years) by body mass index.

|  | **Underweight**  **(n = 20)** | **Normal**  **(n = 271)** | **Overweight**  **(n = 109)** | **Obesity**  **(n = 33)** | **Two-way ANOVA** | **Two-way**  **ANCOVA** |
| --- | --- | --- | --- | --- | --- | --- |
| Grains | 45.88 | 39.16 | 38.99 | 35.41 | NS | NS |
| Grains and flours | 3.85 | 4.54 | 2.89 _b_ | 2.67 | BMI * | NS |
| Bread | 21.66 | 19.59 | 20.45 | 16.91 | NS | NS |
| Breakfast cereals and cereal bars | 1.90 | 1.65 | 1.62 | 1.73 | NS | NS |
| Pasta | 6.04 | 4.92 _a_ | 4.17 _b_ | 4.49 | S** BMI**I* | S** BMI* I* |
| Bakery and pastry | 12.74 | 9.28 | 10.85 | 10.51 | S*I* | S* I* |
| Vegetables | 17.72 | 21.02 | 20.62 | 23.93 | NS | NS |
| Fruits | 12.08 | 18.68 | 18.59 | 20.83 | NS | NS |
| Oils and fats | - | - | - | - |  |  |
| Milk and dairy products | 0.20 | 0.31 | 0.22 | 0.33 | NS | NS |
| Yogurt and fermented milk | 0.00 | 0.21 | 0.11 | 0.30 | NS | NS |
| Other dairy products | 0.20 | 0.10 | 0.11 | 0.03 | NS | NS |
| Fish and Shellfish | - | - | - | - |  |  |
| Meat and meat products | - | - | - | - |  |  |
| Eggs | - | - | - | - |  |  |
| Pulses | 10.68 | 9.25 | 9.17 | 7.28 | NS | NS |
| Sugars and sweets | 3.16 | 1.07 | 1.16 | 0.71 | S* | NS |
| Chocolates | 2.35 | 0.77 | 0.82 | 0.46 | NS | NS |
| Jams and other | 0.82 | 0.23 | 0.26 | 0.06 | NS | NS |
| Other sweets | 0.00 | 0.07 | 0.09 | 0.20 | NS | NS |
| Appetizers | 1.83 | 2.03 | 2.73 | 3.47 | NS | NS |
| Ready-to-eat-meals | 6.12 | 4.81 | 5.18 | 5.28 | NS | NS |
| Sauces and condiments | 1.64 | 2.15 | 1.83 | 1.27 | NS | NS |
| Non-alcoholic beverage | 0.36 | 0.59 | 0.43 | 0.60 | NS | NS |
| Juices and nectars | 0.36 | 0.53 | 0.39 | 0.27 | NS | NS |
| Other drinks | 0.00 | 0.06 | 0.04 | 0.33 | NS | NS |
| Supplements and meal replacers | 0.00 | 0.12 | 0.09 | 0.00 | NS | NS |

Two-way ANOVA was performed taking into account sex (S) and body mass index (BMI); Two-way ANCOVA was performed taking into account S and BMI and age and physical activity as covariates; Two way ANOVA significant differences between BMI classification: a: regarding underweight, b: regarding normal weight, c: regarding overweight; I: Interaction; * *p* < 0.05, ** *p* < 0.01. NS: non-significant.

**Table S3.** Dietary food sources of total fibre (%) in the whole sample of the ANIBES Study Spanish adult population (18–64 years) by the presence or absence of abdominal obesity using the waist to height ratio.

|  | **Non-abdominal obesity**  **(n = 689)** | **Abdominal obesity**  **(n = 966)** | **Two-way ANOVA** | **Two-way ANCOVA** |
| --- | --- | --- | --- | --- |
| Grains | 40.56 | 38.12 | S*WHtR** | NS |
| Grains and flours | 3.76 | 3.24 | NS | NS |
| Bread | 20.80 | 21.18 | S** | S* WHtR* |
| Breakfast cereals and cereal bars | 1.94 | 1.24 | WHtR** | S* |
| Pasta | 6.45 | 6.16 | S** | WHtR* |
| Bakery and pastry | 8.37 | 7.24 | NS | S* |
| Vegetables | 22.89 | 25.08 | S*WHtR* | NS |
| Fruits | 15.78 | 17.18 | S*I** | WHtR*** I** |
| Oils and fats | - | - |  |  |
| Milk and dairy products | 0.37 | 0.41 | NS | NS |
| Yogurt and fermented milk | 0.26 | 0.36 | NS | NS |
| Other dairy products | 0.10 | 0.05 | NS | NS |
| Fish and Shellfish | - | - |  |  |
| Meat and meat products | - | - |  |  |
| Eggs | - | - |  |  |
| Pulses | 8.92 | 9.53 | NS | NS |
| Sugars and sweets | 0.84 | 0.56 | S*WHtR* | S* WHtR* |
| Chocolates | 0.62 | 0.32 | S*WHtR** | S* WHtR* |
| Jams and other | 0.17 | 0.18 | NS | NS |
| Other sweets | 0.05 | 0.06 | NS | NS |
| Appetizers | 1.53 | 1.53 | NS | NS |
| Ready-to-eat meals | 5.33 | 3.90 | S** WHtR*** | NS |
| Sauces and condiments | 2.36 | 2.05 | S**WHtR* | S** |
| Non-alcoholic beverage | 0.54 | 0.45 | NS | NS |
| Juices and nectars | 0.50 | 0.42 | NS | NS |
| Other drinks | 0.04 | 0.04 | NS | NS |
| Supplements and meal replacers | 0.09 | 0.18 | NS | NS |

Two-way ANOVA was performed taking into account sex (S) and the waist to height ratio (WHtR); Two-way ANCOVA was performed taking into account S and the WHtR and the age and physical activity as covariates. I: interaction. * *p* < 0.05. ** *p* < 0.01. *** *p* < 0.001. NS: non-significant.

**Table S4.** Dietary food sources of total fibre (%) in the plausible reporters of the ANIBES Study Spanish adult population (18–64 years) by the presence or absence of abdominal obesity using the waist to height ratio.

|  | **Non-abdominal obesity**  **(n = 263)** | **Abdominal obesity**  **(n = 170)** | **Two-way ANOVA** | **Two-way ANCOVA** |
| --- | --- | --- | --- | --- |
| Grains | 40.30 | 37.35 | NS | NS |
| Grains and flours | 4.40 | 3.27 | NS | NS |
| Bread | 19.96 | 19.30 | NS | NS |
| Breakfast cereals and cereal bars | 1.73 | 1.55 | NS | NS |
| Pasta | 5.20 | 4.05 | WHtR* | NS |
| Bakery and pastry | 9.86 | 10.04 | S* | S* |
| Vegetables | 20.79 | 21.30 | S* | S* |
| Fruits | 17.71 | 19.75 | NS | NS |
| Oils and fats | - | - |  |  |
| Milk and dairy products | 0.30 | 0.26 | NS | NS |
| Yogurt and fermented milk | 0.18 | 0.18 | NS | NS |
| Other dairy products | 0.12 | 0.08 | NS | NS |
| Fish and Shellfish | - | - |  |  |
| Meat and meat products | - | - |  |  |
| Eggs | - | - |  |  |
| Pulses | 8.66 | 9.89 | NS | NS |
| Sugars and sweets | 1.27 | 0.99 | NS | NS |
| Chocolates | 0.94 | 0.66 | NS | NS |
| Jams and other | 0.26 | 0.23 | NS | NS |
| Other sweets | 0.07 | 0.09 | NS | NS |
| Appetizers | 2.11 | 2.60 | NS | NS |
| Ready-to-eat meals | 5.06 | 4.90 | NS | WHtR** |
| Sauces and condiments | 2.23 | 1.59 | NS | NS |
| Non-alcoholic beverage | 0.61 | 0.43 | NS | NS |
| Juices and nectars | 0.54 | 0.35 | NS | NS |
| Other drinks | 0.06 | 0.09 | NS | NS |
| Supplements and meal replacers | 0.11 | 0.08 | NS | NS |

Two-way ANOVA was performed taking into account sex (S) and the waist to height ratio (WHtR); Two-way ANCOVA was performed taking into account S and the WHtR and the age and physical activity as covariates. I: interaction. * *p* < 0.05. ** *p* < 0.01. *** *p* < 0.001. NS: non-significant.

**Table S5.** Dietary food sources of total fibre (%) in the whole sample of the ANIBES study of the Spanish adult population (18­–64 years) by the presence or absence of excess body weight and/or abdominal obesity using the body mass index and the waist to height ratio.

|  | **Non-**excess body weight and/or abdominal obesity  **(n = 597)** | Excess body weight and/or abdominal obesity  **(n = 1058)** | **Two way**  **ANOVA** | **Two way**  **ANCOVA** |
| --- | --- | --- | --- | --- |
| Grains | 40.43 | 38.40 | BMI- WHtR*I* | I* |
| Grains and flours | 3.78 | 3.28 | NS | NS |
| Bread | 20.48 | 21.33 | S* | S* BMI- WHtR* |
| Breakfast cereals and cereal bars | 2.01 | 1.26 | BMI- WHtR** | NS |
| Pasta | 6.40 | 6.21 | S* | BMI- WHtR* |
| Bakery and pastry | 8.49 | 7.27 | S* | S* |
| Vegetables | 22.77 | 24.96 | S*BMI- WHtR* | NS |
| Fruits | 16.23 | 16.81 | I* | BMI- WHtR***I** |
| Oils and fats | - | - |  |  |
| Milk and dairy products | 0.36 | 0.41 | NS | NS |
| Yogurt and fermented milk | 0.27 | 0.35 | NS | NS |
| Other dairy products | 0.09 | 0.06 | NS | NS |
| Fish and Shellfish | - | - |  |  |
| Meat and meat products | - | - |  |  |
| Eggs | - | - |  |  |
| Pulses | 8.99 | 9.44 | NS | NS |
| Sugars and sweets | 0.84 | 0.58 | S* I* | S** |
| Chocolates | 0.61 | 0.35 | S*BMI- WHtR* I* | S* I* |
| Jams and other | 0.18 | 0.18 | NS | NS |
| Other sweets | 0.04 | 0.06 | NS | NS |
| Appetizers | 1.57 | 1.50 | NS | NS |
| Ready-to-eat-meals | 5.04 | 4.19 | S*BMI- WHtR* | NS |
| Sauces and condiments | 2.39 | 2.06 | S*BMI- WHtR* | S* |
| Non-alcoholic beverage | 0.55 | 0.45 | NS | NS |
| Juices and nectars | 0.52 | 0.41 | NS | NS |
| Other drinks | 0.03 | 0.04 | NS | NS |
| Supplements and meal replacers | 0.10 | 0.16 | NS | NS |

Two-way ANOVA was performed, taking into account sex (S) and the body mass index and/or waist to height ratio (BMI-WHtR); Two-way ANCOVA was performed taking into account S and the BMI-WHtR and the age and physical activity as covariates. I: interaction. * *p* < 0.05. ** *p* < 0.01. *** *p* < 0.001. NS: non-significant.

**Table S6.** Dietary food sources of total fibre (%) in the plausible reporters of the ANIBES study of the Spanish adult population (18­–64 years) by the presence or absence of excess body weight and/or abdominal obesity using the body mass index and the waist to height ratio.

|  | **Non-**excess body weight and/or abdominal obesity  **(n = 248)** | Excess body weight and/or abdominal obesity  **(n = 185)** | **Two way**  **ANOVA** | **Two way**  **ANCOVA** |
| --- | --- | --- | --- | --- |
| Grains | 40.22 | 37.70 | NS | I* |
| Grains and flours | 4.50 | 3.21 | BMI- WHtR* | NS |
| Bread | 19.72 | 19.67 | I* | I* |
| Breakfast cereals and cereal bars | 1.69 | 1.63 | NS | NS |
| Pasta | 5.20 | 4.14 | BMI- WHtR* | NS |
| Bakery and pastry | 9.86 | 10.02 | S* | S* I* |
| Vegetables | 20.74 | 21.33 | S* I* | S* |
| Fruits | 17.77 | 19.51 | NS | BMI- WHtR* |
| Oils and fats | - | - |  |  |
| Milk and dairy products | 0.31 | 0.25 | NS | NS |
| Yogurt and fermented milk | 0.19 | 0.17 | NS | NS |
| Other dairy products | 0.12 | 0.07 | NS | NS |
| Fish and Shellfish | - | - |  |  |
| Meat and meat products | - | - |  |  |
| Eggs | - | - |  |  |
| Pulses | 8.81 | 9.60 | NS | NS |
| Sugars and sweets | 1.26 | 1.03 | NS | NS |
| Chocolates | 0.90 | 0.73 | NS | NS |
| Jams and other | 0.28 | 0.21 | NS | NS |
| Other sweets | 0.07 | 0.09 | NS | NS |
| Appetizers | 2.16 | 2.50 | NS | NS |
| Ready-to-eat-meals | 5.01 | 4.98 | NS | BMI- WHtR** |
| Sauces and condiments | 2.23 | 1.64 | NS | NS |
| Non-alcoholic beverage | 0.63 | 0.41 | NS | NS |
| Juices and nectars | 0.57 | 0.33 | BMI- WHtR* | NS |
| Other drinks | 0.07 | 0.08 | NS | NS |
| Supplements and meal replacers | 0.11 | 0.07 | NS | NS |

Two-way ANOVA was performed, taking into account sex (S) and the body mass index and/or waist to height ratio (BMI-WHtR); Two-way ANCOVA was performed taking into account S and the BMI-WHtR and the age and physical activity as covariates. I: interaction. * *p* < 0.05. ** *p* < 0.01. *** *p* < 0.001. NS: non-significant
